# Recent Advances and Challenges in Ammonia‐Hydrogen Energy Conversion

**DOI:** 10.1002/advs.75572

**Published:** 2026-05-07

**Authors:** Menghao Lv, Runchao Qin, Jianshuo Chen, Kaiwen Yang, Jianwei Lu, Weiwei Lei, Yifu Yu

**Affiliations:** ^1^ Institute of Molecular Plus School of Science Tianjin University Tianjin China; ^2^ National Industry−Education Platform for Energy Storage Tianjin University Tianjin China; ^3^ School of Science RMIT University STEM College Melbourne Melbourne Victoria Australia; ^4^ State Key Laboratory of Synthetic Biology Tianjin University Tianjin China

**Keywords:** ammonia‐hydrogen conversation, ammonia decomposition, ammonia synthesis, catalysis, renewable energy

## Abstract

Recently, hydrogen energy has emerged as a key pathway to address the change of global climate and the intermittency of renewable energy, demonstrating significant strategic value in new energy storage systems. However, hydrogen is required to be liquefied at extremely low temperatures or stored in gaseous form under high pressure. Both methods are energy‐consuming and difficult to operate. Ammonia (NH_3_) is recognized as an ideal hydrogen carrier due to its high hydrogen density as well as mature infrastructure for storage and transportation, which has the potential to further reduce the cost of hydrogen utilization. In this context, the integrated ammonia‐hydrogen energy system composed of synthetic ammonia and ammonia decomposition not only contributes to achieving low‐carbon energy transition goals but also supports distributed power generation applications. This review systematically summarizes recent advances and challenges in key technologies for ammonia synthesis and decomposition, providing insights for the development of novel energy storage systems.

## Graphical abstract

This work summarizes the latest progress of various catalytic methods in ammonia‐hydrogen energy conversion. The challenges and outlook of ammonia‐hydrogen energy conversion system are also emphasized. This work expects to build a blueprint of renewable and efficient ammonia‐hydrogen energy conversion systems under mild conditions.

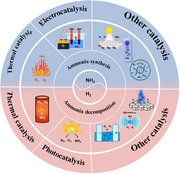

## 1 Introduction

To mitigate global warming caused by the extensive use of fossil fuels, accelerating the transition of current energy structure toward clean energy has become a critical solution [1]. The renewable energy sources including hydropower, wind power and solar power have emerged as major pathways for energy transition, due to their environmental friendliness and renewability. However, their large‐scale deployment is hindered by the intermittency, fluctuation, and high costs [2]. Therefore, the development of novel energy storage technologies is essential to address these challenges. Hydrogen energy storage is widely regarded as a highly flexible secondary energy source for large‐capacity and long‐duration storage. Specifically, hydrogen possesses a high combustion heat value of ∼120 MJ kg^−1^, which is approximately three times that of gasoline for power propulsion. And hydrogen combustion only produces water and generates no carbon dioxide during the process. Thus, it achieves zero‐carbon emissions and enhances economic viability [3, 4, 5]. However, the inherent characteristics including low volumetric energy density, low boiling point and flammability have hindered its large‐scale application, leading to challenges in storage, transportation and safety [6]. Consequently, developing mature, safe, and efficient hydrogen storage and transportation technologies is urgently required.

NH_3_ stands out among many hydrogen carriers such as methanol, methane, formic acid, and related compounds or hydrides, due to its distinct advantages in safety, storage, and transport [7, 8]. First, as a carbon‐free carrier, its decomposition only produces H_2_ and N_2_ without carbon by‐products, while methanol and methane decomposition generates CO/CO_2_ requiring additional carbonization. Second, the strong pungent smell of NH_3_ is easily detectable even at low concentrations (5 ppm). This provides a natural advantage for the early detection of ammonia leakage. Meanwhile, ammonia features a narrow flammable limit (15.5%∼28%) and high ignition point, plus a pungent odor detectable at 5 ppm, making its safety risk much lower than methanol and hydrogen. Moreover, the energy density of ammonia is 15.6 MJ L^−1^, which is significantly higher than that of liquid hydrogen, methanol and so on. Notably, the liquefaction temperature of ammonia is –33°C under ambient pressure, compared to –253°C for hydrogen. These properties collectively reduce energy consumption for storage, lower infrastructure investment, and enhance security [9, 10]. In addition, the easy liquefaction feature of ammonia gas enables it to be seamlessly integrated with existing transportation systems, including road tankers, railway tankers and ocean‐going vessels, thereby eliminating the need for new infrastructure and promoting large‐scale and long‐distance logistics [11, 12, 13, 14].

At present, ammonia synthesis in industry mainly relies on the Haber‐Bosch (H‐B) process at high temperatures (300°C–400°C) and pressures (15–30 MPa). The strict operating conditions result in its annual energy consumption accounting for approximately 2% of the global total energy consumption [15]. Meanwhile, the hydrogen used in this process mainly comes from natural gas steam reforming and coal gasification, emitting approximately 450 million tons of carbon dioxide (CO_2_) annually, accounting for about 1.3% of the global total CO_2_ emissions [16, 17, 18, 19]. The ammonia synthesized through the traditional Haber process mentioned above is referred to as gray ammonia, whereas the ammonia produced using green hydrogen as the raw material (green hydrogen is produced by electrolyzing water using renewable energy sources such as wind power, photovoltaics, and hydropower) is known as green ammonia. The entire production process has nearly zero carbon emissions and has received widespread attention in recent years.

Meanwhile, for hydrogen production through ammonia decomposition, the theoretical equilibrium conversion rate at 400°C can reach 99%. However, due to the limitations of reaction kinetics, without the use of a catalyst, the reaction energy barrier can only be overcome at a temperature of 500°C to 800°C. Therefore, the development of green and efficient ammonia synthesis and ammonia decomposition technologies under mild conditions plays a crucial role in the sustainable development of ammonia‐hydrogen energy conversion system [20].

**FIGURE 1:**
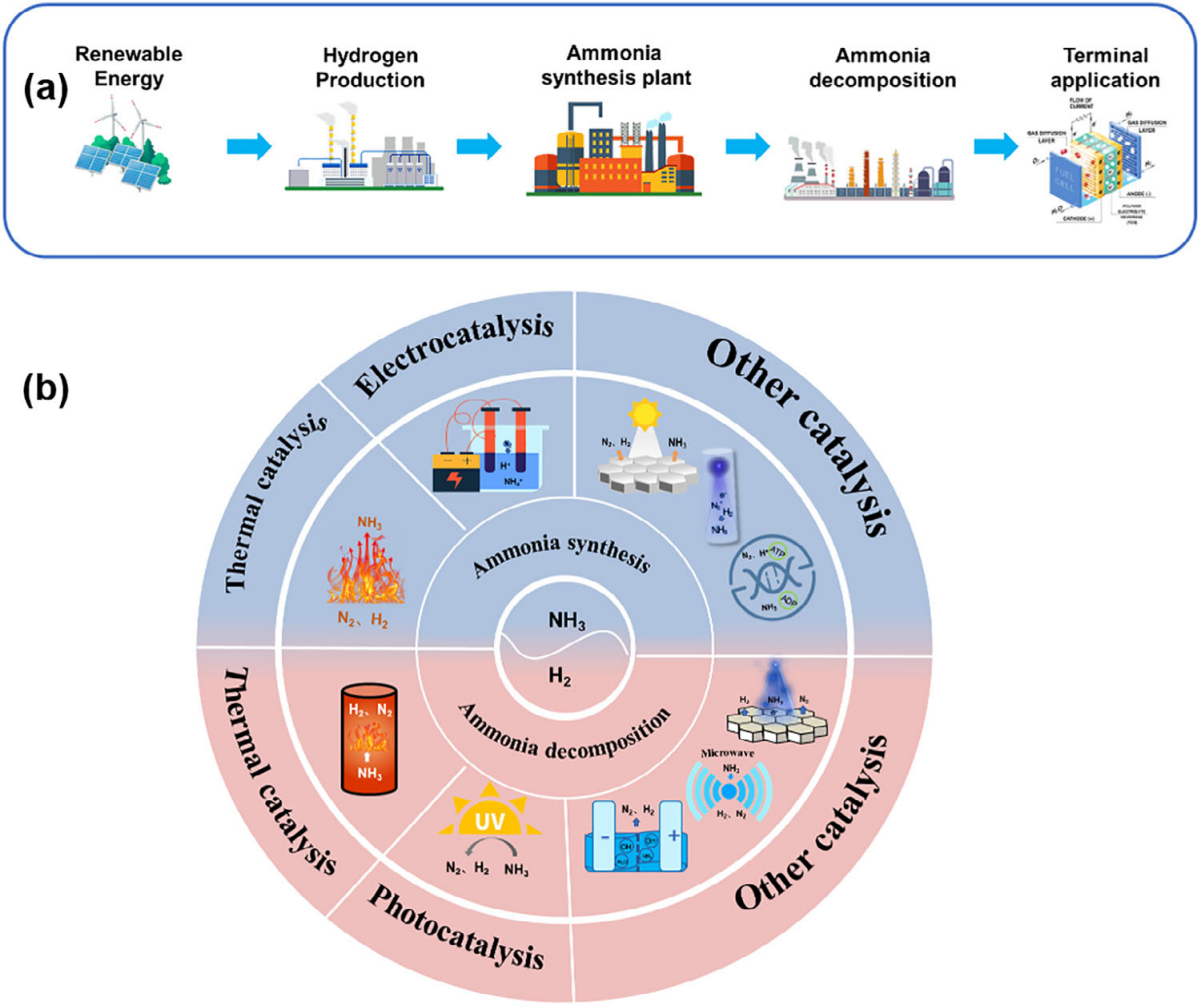
(a) Ammonia‐hydrogen energy conversion system. (b) Various methods for ammonia synthesis and decomposition under mild conditions.

In recent years, extensive exploration has been conducted on efficient ammonia‐hydrogen energy conversion system under mild conditions, including ammonia synthesis and ammonia decomposition technologies (Figure 1a). This article summarizes the latest progress of various catalytic methods, including thermal catalysis, photocatalysis, electrocatalysis and so on. Their mechanisms, catalyst designs and performances are individually presented in form of formulas and figures to directly deliver the innovation points and advantages with their design strategies, which can significantly facilitate understanding of the corresponding design idea (Figure 1b). Finally, the challenges and outlook of ammonia‐hydrogen energy conversion system are also emphasized. This work expects to build a blueprint of renewable and efficient ammonia‐hydrogen energy conversion systems under mild conditions.

## 2 Ammonia Synthesis

### 2.1 Thermal Catalytic Ammonia Synthesis From Nitrogen

#### 2.1.1 Synthesis Mechanism

The N≡N triple bond in N_2_ molecule exhibits exceptional stability, characterized by a substantial bond dissociation energy of 941 kJ mol^−1^, which renders it chemically inert under standard conditions [21]. Accordingly, the Haber‐Bosch process functions at high temperatures (300°C–500°C) and pressures (150–200 atm), utilizing catalysts like Fe_2_O_3_‐Al_2_O_3_‐K_2_O [22, 23]. The overall reaction can be represented by (Equation (1)): [24]

(1) $$N_{2}+3H_{2}\;⇌\;2NH_{3},\;\Delta_{f}H_{0}=-94.2\;\mathrm{kJ}\;\mathrm{mo}l^{-1}$$

**FIGURE 2:**
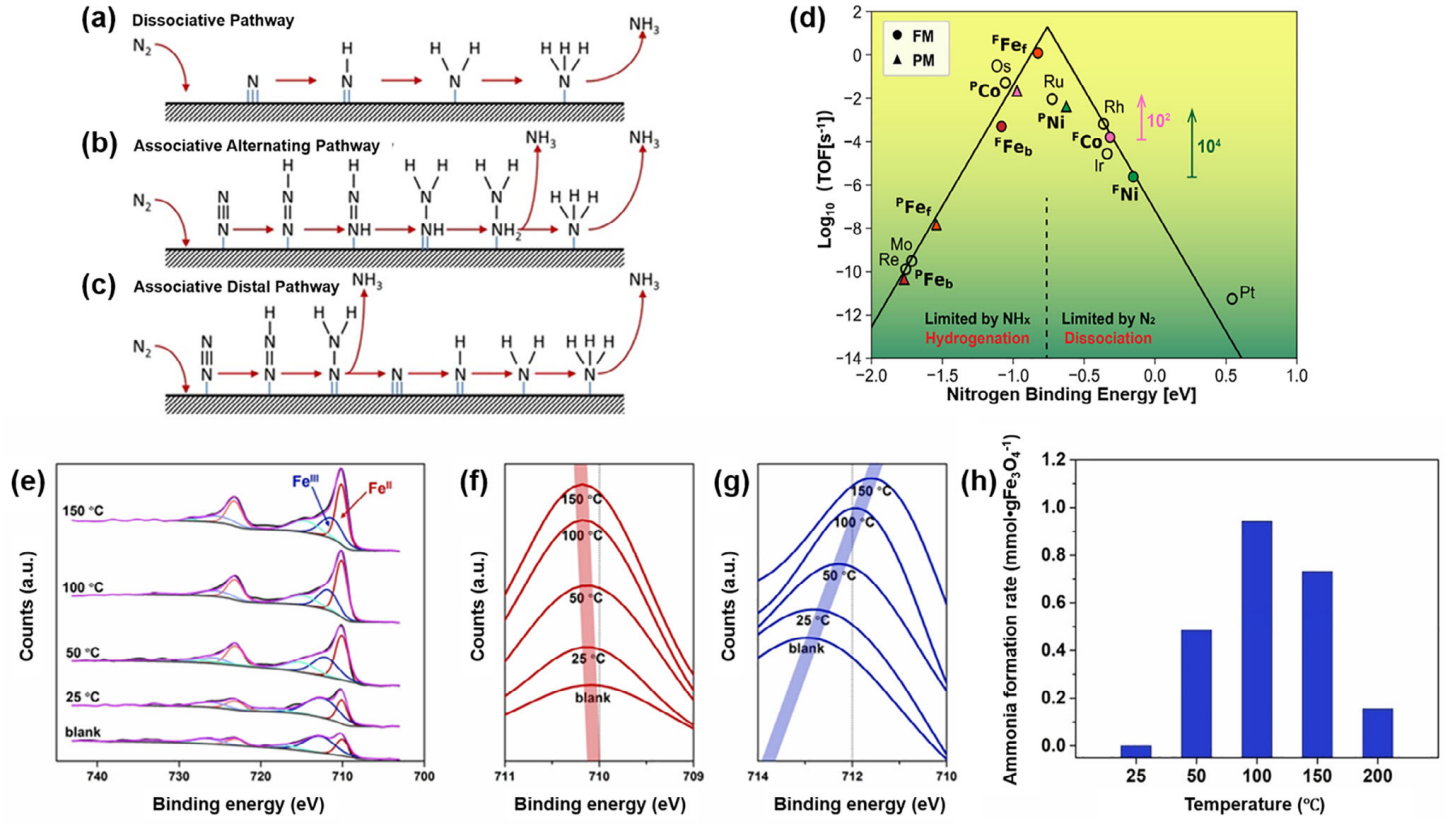
(a–c) Generic mechanisms for nitrogen reduction to ammonia on heterogeneous catalysts. Reproduced with permission [26]. Copyright 2026, ELSEVIER. (d) Computationally established Sabatier principle for ammonia synthesis activity on the step sites of different transition metals. The calculations of bcc Fe (Fe_b_), fcc Fe (Fe_f_), hcp Co, and fcc Ni are shown with orange, dark red, pink, and green markers, respectively. The ferromagnetic states (^F^M) of these metals are shown as solid points, and the paramagnetic states (^P^M) are shown as solid triangles. All other transition metals are shown with black empty circles. Reproduced with permission [34]. Copyright 2026, American Chemical Society. (e) In situ XPS spectra at Fe _2p_ of Fe_3_O_4_@MIL‐101(Fe) at different temperature under 1 MPa N_2_ and H_2_ mixture (N_2_ 25 %, H_2_ 75 %) with the condition of 100°C under vacuum environment as blank. (f) The amplifying image of the XPS spectra at Fe^II^ 2p_1/2_ orbitals. (g) The amplifying image of the XPS spectra at Fe^III^ 2p_1/2_ orbitals. (h) NH_3_ formation rate at different temperature (1 MPa, 6 h). Reproduced with permission [38]. Copyright 2026, WILEY.

Nitrogen reduction reaction (NRR) on heterogeneous catalyst surfaces is typically believed to occur through two mechanistic pathways: the dissociative pathway and the associative pathway (Figure 2a–c) [25, 26]. In the dissociative pathway, N_2_ adsorbs on the catalyst surface and directly cleaves the N≡N triple bond, producing atomic nitrogen species. The surface‐bound nitrogen atoms are subsequently hydrogenated in a stepwise manner to produce NH_x_(ads) intermediates, which finally desorb as ammonia gas (NH_3_(g)). The dissociation of the N≡N bond is significantly energy‐demanding, and the majority of research indicates that the traditional Haber‐Bosch process adheres to this mechanism [27, 28, 29].

The associative pathway can be further classified into distal and alternating pathways. In the associative distal pathway, protonation predominantly occurs on the distal nitrogen atom. In contrast, in the associative alternating pathway, protons are successively supplied to both nitrogen atoms in N_2_. In the associative pathway, the N_2_ molecule experiences successive hydrogenation until the liberation of an NH_3_ molecule coincides with the dissociation of the N‐N bond, necessitating considerably lesser energy [30, 31].

#### 2.1.2 Catalysts

##### 2.1.2.1 Non‐Noble Metal Catalysts

Advanced theoretical studies have assessed the feasibility of ammonia synthesis under mild conditions on transition metal surfaces, uncovering a scaling link between the activation energy for N_2_ dissociation and the adsorption energies of critical intermediates [32, 33]. The classical volcano plot delineates the structure‐activity relationship between catalytic activity for ammonia synthesis and *N adsorption strength: Fe and Ru, with nitrogen binding energies proximate to the volcano apex, demonstrate elevated activity for NH_3_ synthesis, whereas other transition metals (e.g., Co, Ni, Mo) are conventionally regarded as less efficacious due to excessively strong or weak *N adsorption. This is due to that a moderate *N binding energy in ammonia synthesis not only affords sufficient activation energy for the dissociation of the N≡N triple bond, ensuring the efficient cleavage of N_2_, but also neither results in the accumulation of *N on metallic active sites (and the subsequent hindrance of hydrogenation steps) owing to overly strong adsorption nor causes the rapid desorption of *N (and the failure of effective hydrogenation) due to excessively weak adsorption. In contrast, metals such as Co and Ni display overly strong *N adsorption, which readily induces active site poisoning and impedes the hydrogenation of reaction intermediates, while metals like Mo exhibit excessively weak *N adsorption, thus precluding the effective dissociative activation of N_2_. Notably, Wang et al. found that cobalt (Co) and nickel (Ni) experience a Curie phase transition from ferromagnetic to paramagnetic phases, leading to a 2–4 orders of magnitude increase in ammonia synthesis activity, which is ascribed to electronic structure optimization induced by the phase transition that lowers nitrogen adsorption barriers, changing the volcano plot (Figure 2d) [34]. Kojima et al. developed a molybdenum nitride‐based cobalt catalyst (Co_3_Mo_3_N) that achieved an ammonia synthesis rate of 986 µmol h^−1^ m^−2^ under 673 K and 0.1 MPa, significantly outperforming the conventional Fe‐K_2_O‐Al_2_O_3_ system [35]. This breakthrough demonstrates that metals located on the volcano plot can dynamically modulate nitrogen adsorption energy through structural modifications, thereby overcoming intrinsic activity limitations. Furthermore, non‐noble metal composite catalysts, exemplified by Cobalt‐based catalyst supported on Mo_2_CT_x_ MXene (1‐Co_Nit_‐MO_2_CT_x_), achieved a synthesis rate of 9499 µmol g^−1^ h^−1^ under 400°C at ambient pressure, whereas nickel‐based supported on Mo_2_CT_x_ MXene catalyst(Ni/Mo_2_CT_x_)achieved 21.5 mmol g^−1^ h^−1^ at 400°C and 1 MPa, thereby reinforcing the applicability of this approach [36, 37]. These advancements not only broaden the framework of volcano plot theory but also provide multifaceted strategies for the design of innovative high‐performance ammonia synthesis catalysts—via chemical modification, phase transition regulation, or interface engineering—to surpass conventional adsorption energy limitations and create new catalytic systems with remarkable activity [39]. Iron has been intensively researched as a conventional catalyst for ammonia synthesis throughout the past century [40, 41, 42]. In the emerging domain of ammonia synthesis under mild conditions, iron persists as a viable choice owing to its affordability and durability. Cheng et al. created a synergistic catalyst aimed at enhancing product desorption efficiency by uniformly embedding Fe_3_O_4_ particles within the MIL‐101(Fe) metal‐organic framework, resulting in a catalyst featuring three active Fe sites—Fe_3_O_4_@MIL‐101(Fe) [38]. In situ XPS (Figure 2e–g) demonstrated that Fe^3+^ on the surface of Fe_3_O_4_ was gradually reduced to Fe^2+^ by H_2_ as the reaction temperature rose. Fe^II^, with a greater number of outer electrons, was subsequently employed to activate N_2_, in order facilitating the concurrent activation of both H_2_ and N_2_. Simultaneously, Fe^III^ in MIL‐101(Fe) transferred NH_3_ from the active sites, promoting product desorption and the regeneration of catalytic sites. The NH_3_ production rate of the Fe_3_O_4_@MIL‐101(Fe) reached 0.95 mmol g^−1^ Fe_3_O_4_ h^−1^ at 100°C and 1 MPa (Figure 2h). This study illustrated the viability of employing economical Fe‐based catalysts for large‐scale ammonia production under moderate circumstances.

##### 2.1.2.2 Noble Metal Catalysts

Ru‐based catalysts are recognized as the second‐generation catalysts for ammonia synthesis, as they have achieved dual breakthroughs in both catalytic performance and process compatibility relative to the first‐generation Fe‐based catalysts. Their *N binding energy is far more commensurate with the optimal apex of the ammonia synthesis volcano plot, endowing them with markedly superior intrinsic catalytic activity over Fe‐based counterparts; this allows Ru‐based catalysts to deliver high ammonia synthesis rates under milder temperature and pressure conditions, thereby drastically reducing the energy consumption and equipment requirements for industrial‐scale ammonia production [43]. Notably, many ruthenium‐based catalysts reported in recent years, such as Ru/CeO_2_ [44], Ru/C12A7:e^−^ [45], and Ru/Ba‐Ca(NH_2_)_2_ [46], exhibit high activity in ammonia synthesis, while suffering from the problem of easy hydrogen poisoning. Miyahara et al. studied a range of Ru catalysts supported on La_2_O_3_, Pr_2_O_3_, Nd_2_O_3_, Sm_2_O_3_, and Ga_2_O_3_. All displayed greater basicity than MgO at 400°C. Reports indicate that lanthanide oxides may substantially improve the stability of Ru‐based catalysts. The supports, possessing enhanced electron‐donating capabilities, efficiently promoted the H_2_ dissociation process, providing a near‐zero reaction order of H_2_ and markedly mitigating hydrogen poisoning in comparison to Ru/MgO. Among these catalysts, Ru/Pr_2_O_3_ exhibited superior performance, attaining an ammonia synthesis rate of 49 mmol g^−1^ h^−1^ at 400°C and 1 MPa, which was 12 times more than that of Ru/MgO under same conditions [47]. Osozawa et al. formulated the optimal Ru/Cs^+^/CeO_2_, configuration, yielding ammonia synthesis rates of 0.55, 4.62, and 5.23 mmol h^−1^ g^−1^_cat._ at 300°C, 350°C, and 400°C, respectively. Notably, CeO_2_ support itself features the Ce^3+^/Ce^4+^ redox couple and oxygen vacancy structures, which can act as an electronic channel to facilitate electron transfer from the promoter Cs^+^ to Ru active sites, thereby further amplifying the electron donation effect. Furthermore, the synergy between the surface basicity and oxygen vacancies of CeO_2_ weakens the strong adsorption of H_2_ on Ru active sites, and thus effectively mitigates hydrogen poisoning, as reflected by the variation of the H_2_ reaction order from –0.7 for the reference catalyst to 0.2 [48].

Jiang et al. revealed that the inclusion of fullerene (C_60_) clusters as molecular promoters markedly improved ammonia synthesis rates over both Mo‐based and Ru‐based catalysts across diverse supports [49]. Furthermore, the aberration‐corrected integrated differential phase contrast (AC iDPC) technique, high‐angle annular dark‐field (HAADF) imaging, and energy dispersive X‐ray spectroscopy (EDS) analyses indicate that C_60_ adheres to the matrix surface rather than being incorporated into the matrix surface or interface. At a consistent temperature of 400°C, C_60_ promoted Ru/CeO_2_ achieved the highest ammonia synthesis rate of 63.5 mmol NH_3_ g^−^^1^_cat._ h^−1^ at 1 MPa. This system exhibited remarkable stability at 400°C and 1 MPa during 1006 h of continuous operation, sustaining its activity without substantial reduction in ammonia synthesis rates. The C_60_ clusters may serve as active sites for the adsorption, storage, activation, and spillover of H_2_, so substantially alleviating the hydrogen poisoning effect resulting from significant H_2_ adsorption. Moreover, C_60_ functioned as an electronic buffer, equilibrating the electronic density of transition metal sites, thus enhancing the activation of N_2_ on transition metal surfaces and aiding its future activation and migration at active sites. In light of these findings, the team plans to substitute carbon clusters C_60_ with cost‐effective carbon ash (predominantly consisting of C_60_) and investigate large‐scale preparation methods for Ru‐based catalysts enhanced by carbon ash, aiming to showcase the application of this catalyst in ammonia synthesis under mild reaction conditions.

Alongside altering the composition of supports and co‐catalysts, developing catalysts with varied structures represents a viable approach. Gas clusters including [Li_4_FeH_6_], [Li_5_FeH_7_], [Li_4_RuH_6_], and [Ba_2_RuH_6_] can react with dinitrogen to produce clusters that incorporate —NH_2_ groups [50]. Among them, ruthenium analogs are stable compounds that are relatively straightforward to synthesize, and their catalytic efficacy for ammonia synthesis under mild conditions has been documented by Chen et al. [51, 52]. The reaction involved the terminal chemical adsorption of H_2_ on partially positively charged [RuH_6_], resulting in the bending of the H_2_ molecule toward adjacent Li sites. The direct dissociation of H_2_ was kinetically inhibited, with a predominance of a non‐dissociative activation mechanism. Isotopic H/D exchange tests demonstrated that only lattice hydrogen engaged in the hydrogenation processes of activated H_2_, enabling the transfer of electrons and protons, while gaseous H_2_ restored the diminished surface lattice hydrogen. Robust electrostatic interactions between Li/Ba cations and the transition states of intermediate N_x_H_y_ species (x = 0–2, y = 0–3) at each reaction stage augmented the stability of these intermediates. The dynamic synergy among components in the ternary complex hydrides enabled a multistep reaction mechanism characterized by a narrow energy span and well‐balanced kinetic barriers. The ammonia synthesis rate of the ternary barium–ruthenium complex hydride (Ba_2_RuH_6_/MgO) reached 9500 µmol g^−1^_cat._ h^−1^, surpassing the Ru/MgO, Cs_2_O‐Ru/MgO, and BaO‐Ru/MgO catalysts by factors of 25, 6.3, and 9.5, respectively. Subsequent research by the same group revealed that additional ruthenium complex hydrides, such as AM_n_[RuH_m_] (where AM = Li, Na, K, Ca, or Ba), displayed markedly superior activity compared to the benchmark Cs‐Ru/MgO catalyst. The findings highlighted the significant potential of ternary complex hydrides for use in renewable electricity‐driven small‐scale Haber‐Bosch processes. In addition to ternary complex hydrides, catalysts like Ru/BaREO_2_H (RE = Y, Sc) and Ru/Sm_2_O_3_ have demonstrated significant promise in ammonia synthesis, hence expanding the possibilities for the advancement of advanced catalytic systems [53, 54, 55].

This chapter summarizes several catalysts in Table 1. Recently, a number of innovative low‐temperature and low‐pressure catalysts have evolved, yet iron‐based catalysts remain predominant in industrial ammonia synthesis. Ruthenium‐based systems, as second‐generation catalysts for ammonia synthesis, have shown effective industrial application. Enhancing ruthenium‐based catalysts for thermocatalytic ammonia synthesis under mild circumstances is a primary research goal. Nonetheless, their elevated expense has impeded extensive adoption to date. Consequently, the advancement of economical and highly efficient non‐noble metal catalysts possesses considerable theoretical and practical importance for thermocatalytic ammonia synthesis under mild conditions. To advance novel thermocatalysts for practical applications, two strategies require consideration: first, diminishing the loading of noble metal active components by altering the catalyst's surface structure or creating porous supports; second, further investigating non‐noble metal catalysts via alloying or the incorporation of additional elements.

**TABLE 1:** Summary of some recently developed themocatalysts for ammonia synthesis under ambient conditions.

Catalyst	Reactant	NH_3_ production rate	Condition	Refs.
Co_3_Mo_3_N	N_2_/H_2_	986 (µmol m^−2^ h^−1^)	673 (K)、0.1 (MPa)	[35]
1‐Co_Nit_‐Mo_2_CTx	N_2_/H_2_	9499 (µmol g^−1^ h^−1^)	400 (°C)、0.1 (MPa)	[36]
Ni/Mo_2_CT_x_	N_2_/H_2_	21.5 (mmol g^−1^)	400 (°C)、1 (MPa)	[37]
Fe_3_O_4_@MIL‐101(Fe)	N_2_/H_2_	0.95 (mmol g^−1^ Fe_3_O_4_ h^−1^)	100 (°C)、1 (MPa)	[38]
Ru/Pr_2_O_3_	N_2_/H_2_	49 (mmol g^−1^ h^−1^)	400 (°C)、1 (MPa)	[47]
Ru/Cs^+^/CeO	N_2_/H_2_	5.23 (mmol g^−1^_cat._ h^−1^)	400 (°C)、0.1 (MPa)	[48]
C_60_‐ promoting Ru/CeO_2_	N_2_/H_2_	63.5 (mmol_NH3_ g^−1^_cat._ h^−1^)	400 (°C)、1 (MPa)	[49]
Ba_2_RuH_6_/MgO	N_2_/H_2_	9500 (µmol g^−1^_cat._ h^−1^)	573 (K)、1 (bar)	[51]
Ru/BaScO_2_H	N_2_/H_2_	4.09 (mmol g^−1^ h^−1^)	300 (°C)、0.1 (MPa)	[53]
Ru/BaYO_2_H		2.77 (mmol g^−1^ h^−1^)		
Ru/Sm_2_O_3_	N_2_/H_2_	32 214 (µmol g^−1^_cat._ h^−1^)	400 (°C)、1 (MPa)	[54]

### 2.2 Electrocatalytic Ammonia Synthesis From Nitrogen

#### 2.2.1 Synthesis Mechanism

Numerous researchers have endeavored to synthesis ammonia through clean and sustainable methods with the advancement of renewable energy [56, 57, 58, 59]. Inspired by natural processes, an electrochemical system for ammonia synthesis under mild conditions has been established, utilizing H_2_O or H_2_ as raw materials. In this system, H_2_O is oxidized at the anode to O_2_, electrons, H^+^ or OH^−^, which then react with N_2_ at the cathode to generate NH_3_.

The electrochemical nitrogen reduction reaction (eNRR) presents several advantages compared to the conventional Haber‐Bosch process, such as reduced energy consumption, adjustable reaction conditions, equipment simplicity, and environmental sustainability. This method facilitates the generation of ammonia from variable renewable energy sources under mild conditions, substantially lowering ammonia production costs [60, 61].

Electrocatalytic synthesis of ammonia, akin to thermocatalysis, encompasses two fundamental reaction mechanisms: the associative pathway and the dissociative pathway. In molten salt systems, it proceeds predominantly via a dissociative mechanism. The high‐temperature environment (typically 200°C–800°C) of such molten systems provides sufficient thermal energy to surmount the high dissociation energy barrier of the N≡N triple bond, while the absence of proton solvents and insufficient proton supply in the molten media preclude the stepwise hydrogenation of N_2_ required for the associative pathway, efficiently [62, 63, 64, 65, 66, 67]. While in the electrocatalytic synthesis of ammonia at mild conditions, the binding mechanism plays a pivotal role. It is worth noting that dissociation and association mechanisms are not applicable to all electrocatalytic systems. Abghoui and Skúlason hypothesized a method different from the conventional Mars‐van Krevelen (MvK) pathway for transition metal nitrides (TMNs) as catalysts. Their proposal indicated that lattice nitrogen atoms on TMN catalyst surfaces can be successively hydrogenated to produce NH_3_ molecules, resulting in nitrogen vacancies that are later filled by adsorbed N_2_ molecules, thereby replenishing the lattice nitrogen. The MvK mechanism prioritizes nitrogen vacancy replenishment and catalyst regeneration above NH_3_ adsorption, presenting a new perspective on reaction kinetics, in contrast to the previously mentioned [68].

#### 2.2.2 Catalysts

##### 2.2.2.1 Non‐Noble Metal Catalysts

**FIGURE 3:**
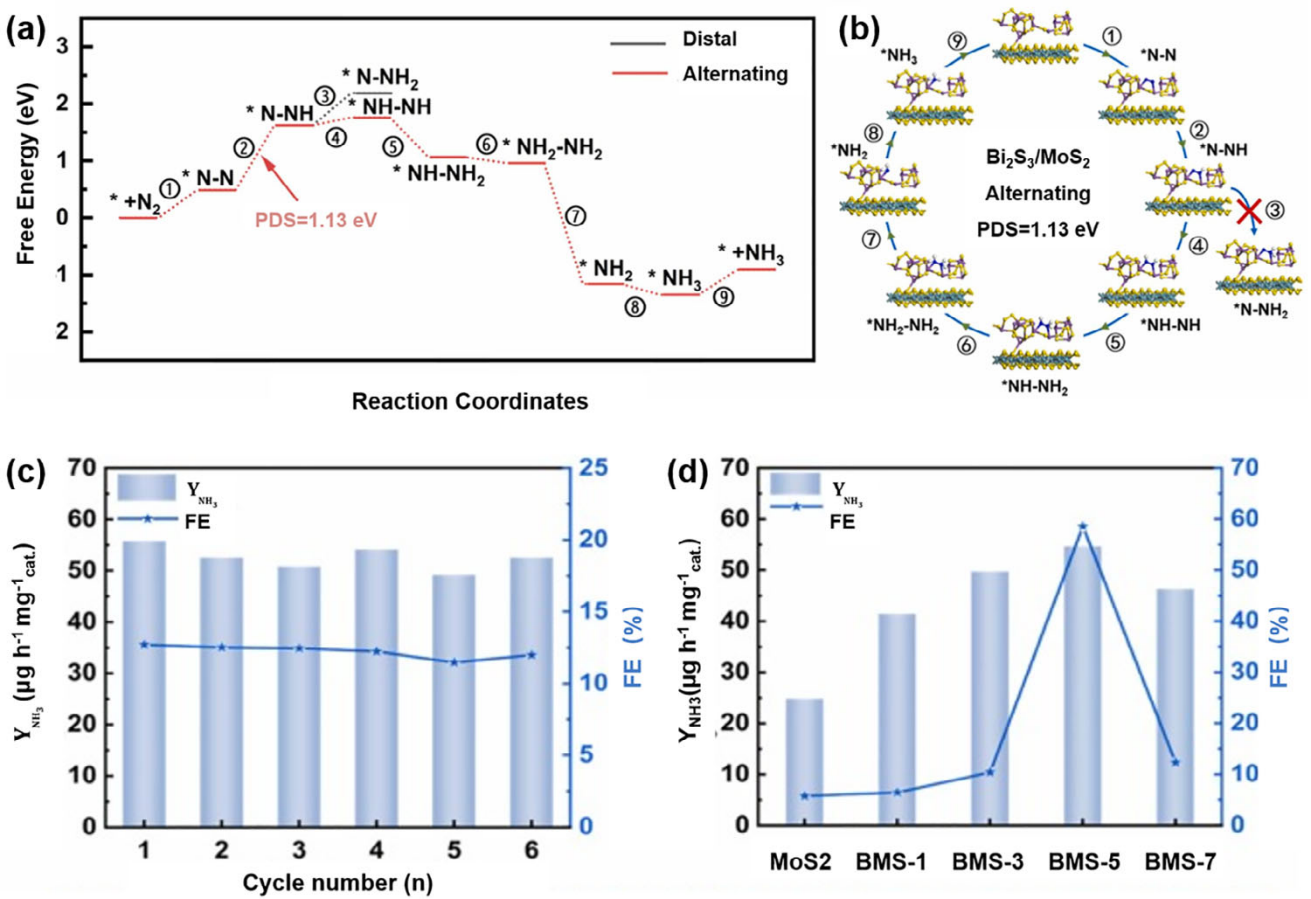
(a) Gibbs free energy diagrams for NRR on Bi_2_S_3_/MoS_2_ composites through distal pathway (black line) and alternating pathway (red line). (b) Schematic diagram of ammonia synthesis by using Bi_2_S_3_/MoS_2_ composites. NH_3_ yield rates and FEs: (c) BMS‐5 at −0.60 V vs. RHE during recycling tests. (d) Bi_2_S_3_/MoS_2_ composites with different Bi content. Reproduced with permission [70]. Copyright 2026, Elsvier.

**FIGURE 4:**
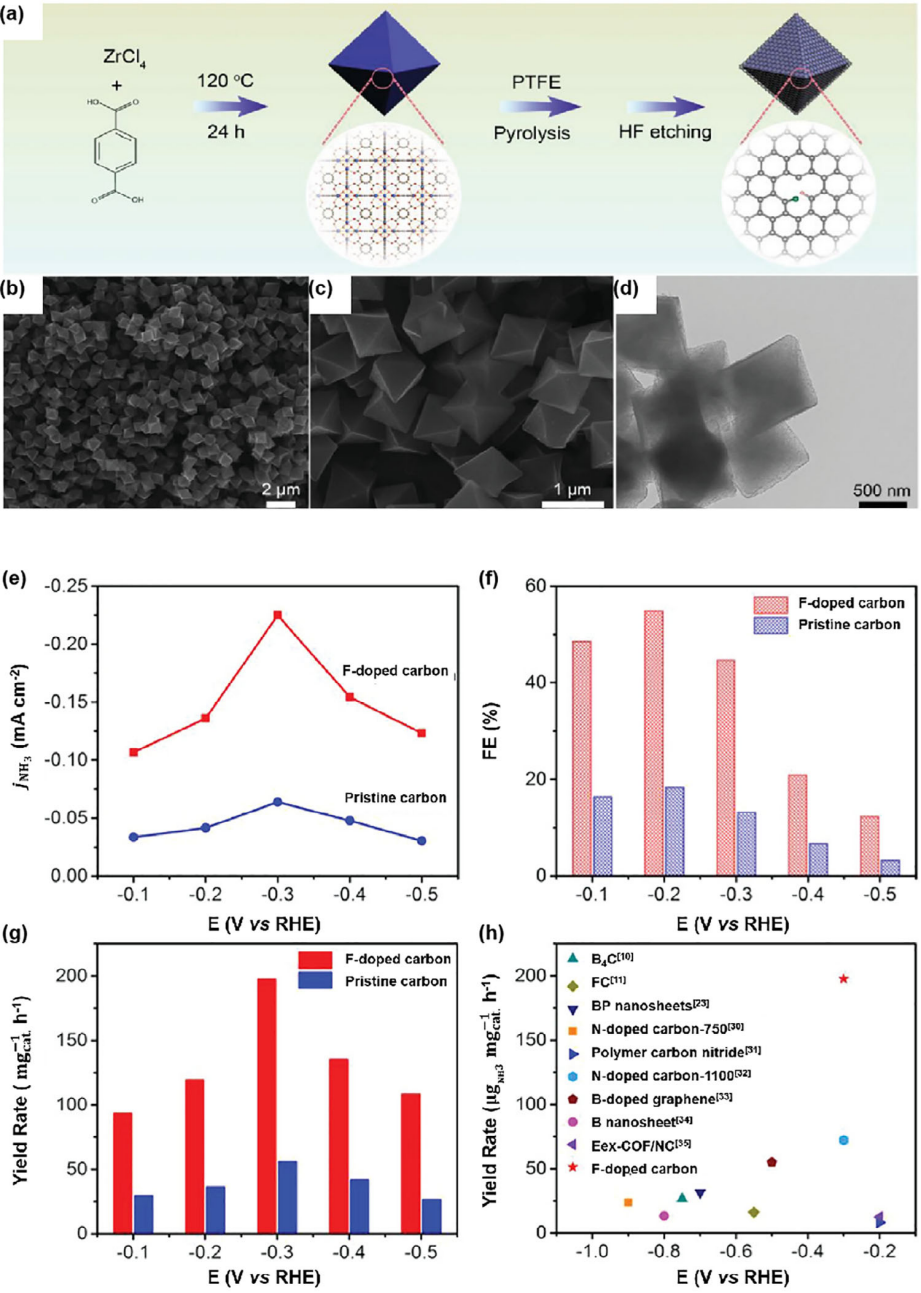
(a) Scheme of the synthetic procedure for F‐doped carbon. (b) A typical SEM image. (c) magnified SEM image. (d) TEM image. (e) Current density for NH_3_ (f) FE, and (g) yield rate for NH_3_ of F‐doped carbon and pristine carbon at different applied potentials. (h) Comparison of the yield rate of NH_3_ over F‐doped carbon with other metal‐free electrocatalysts under near‐ambient conditions. Reproduced with permission [73]. Copyright 2026, Wiley.

Yu et al. produced bismuth nanocrystal catalysts (BiNCs) using an enhanced polyol reduction technique, exhibiting significant nitrogen reduction reaction performance. At –0.60 V vs RHE, BiNCs attained a Faradaic efficiency (FE) of 67% and an effective current density of 0.50 mA cm^−2^, sustaining a consistent FE (67%) and current density during 48 h of continuous reaction. The authors attributed the performance to the robust interaction between the Bi metal 6p band and N 2p orbitals, which stabilized the *NNH intermediate and diminished the energy barrier of the potential‐determining step (PDS). Additionally, the incorporation of potassium ions in the solution facilitated proton‐coupled electron transport, hence reducing *ΔG* and improving reaction selectivity [69]. Similarly, Meng et al. demonstrated Bi's function in inhibiting HER through the synthesis of the composite material Bi_2_S_3_/MoS_2,_ and the calculation showed that the reaction process followed the alternating path (Figure 3a,b). The robust interaction between Bi and N orbitals was confirmed to enhance N_2_ adsorption and activation, leading to increased NRR selectivity. At –0.60 V vs. RHE, the ammonia yield attained 54.64 µg h^−1^ mg^−1^_cat._, while at –0.40 V vs. RHE, the Faradaic efficiency was 58.56%, with no notable performance deterioration seen after 12 h of reaction (Figure 3c,d) [70]. Jiang et al. found that phosphotungstic acid (PTA), characterized by a high density of oxygen sites, effectively anchors multifunctional metal species. Additionally, electrons within the polyanion framework can migrate to these anchored metal species, thereby modulating their electronic structure. By co‐anchoring HER‐suppressing Bi and Li metals onto PTA, a co‐functionalized catalyst, LiBi@VO‐PTA, was developed. At 0.1 V vs. RHE, this catalyst produced an ammonia yield of 61 ± 1 µg h^−1^ mg^−1^_cat._, with a FE of 85 ± 2% [71]. Metal‐free materials offer benefits including low cost, superior stability, and high conductivity. Their structures can be modified by doping with various atoms to create advantageous defects and active sites for reactions [72]. Zeng et al. developed an efficient metal free catalyst by incorporating fluorine (F) atoms into a 3D porous carbon framework, maintaining a regular octahedral shape (Figure 4a–d). The Lewis acid sites generated during this process have unfavorable interactions with protons (H^+^), inhibiting the hydrogen evolution process (HER) and hence improving the selectivity for N_2_ electroreduction to NH_3_. At –0.2 V vs RHE, the fluorine‐doped porous carbon attained a peak Faradaic efficiency (FE) of 54.8%, which is three times greater than that of the unmodified carbon framework (18.3%). At –0.3 V vs RHE, the ammonia production of the F‐doped carbon attained 197.7 µg mg^−1^_cat._ h^−1^ (Figure 4e–h) [73]. Li et al. investigated the NRR performance of composite metal‐free materials. Density functional theory (DFT) calculations revealed that bending strain generated by interfacial hybridization in the catalyst (h‐BNNs/CNTs) modifies the electronic structure of boron nitride, therefore lowering the energy barrier for the eNRR and inhibiting the hydrogen evolution process (HER). In a saturated 0.1 м Na_2_SO_4_ electrolyte, the catalyst attained an ammonia yield of 36.5 µg mg^−1^_cat._ h^−1^ and a FE of 63.9% at –0.691 V vs. RHE [74]. Du et al. innovatively concentrated on a boron‐ and sulfur‐*co*‐doped carbon nanofiber (S‐B/CNFs) system. By meticulously adjusting the pz orbital center position of boron, they effectively mitigated the significant energy barrier related to N_2_ adsorption and activation. The S_6.23_‐B_8.09_/CNFs catalyst attained an ammonia yield of 0.223 µmol cm^−2^ h^−1^ and a FE of 22.4% at –0.7 V vs. RHE in a 0.5 M K_2_SO_4_ electrolyte. Theoretical and experimental investigations demonstrated that boron functions as an electron acceptor, while sulfur and phosphorus work as electron donors, collectively modulating nitrogen reduction reaction activity. The doping of sulfur atoms induced a shift in the pz orbital center of boron atoms, optimizing the p‐band center structure, hence improving N_2_ adsorption at S‐C‐B sites and lowering the energy barrier for the rate‐determining step of N_2_ protonation to generate *NNH.

The S_6.23_‐B_8.09_/CNFs exhibited a plethora of active BC_3_ sites and defective carbon structures, which synergistically enhanced NRR activity. Furthermore, the S_6.23_‐B_8.09_/CNFs exhibited remarkable stability and selectivity, maintaining consistent ammonia yield and Faradaic efficiency in 10 consecutive cycles and 10‐h long‐term electrolysis tests, without the creation of hydrazine. The materials utilized in that study do not include costly metals, guaranteeing affordability and widespread accessibility, which meets the requirements for extensive industrial production, giving substantial economic backing for sustainable development [75]. This chapter summarizes several catalysts in Table 2.

**TABLE 2:** Summary of some recently developed electrocatalysts for ammonia synthesis under ambient conditions.

Catalyst	Electrolyte	Applied potential [V vs RHE]	Reactant	NH_3_ production rate	Faradaic efficiency (%)	Refs.
BiNCs	1 м K^+^, pH=3.5	−0.70	N_2_、H_2_O	200 (mmol g^−1^ h^−1^)	67	[69]
Bi_2_S_3_/MoS_2_	0.1 м Na_2_SO_4_	−0.60	N_2_、H_2_O	54.64 (µg mg^−1^_cat._ h^−1^)	58.56	[70]
LiBi@V_O_‐PTA	0.1 м Li_2_SO_4_	−0.1	N_2_、H_2_O	61 ± 1 (µg mg^−1^_cat._ h^−1^)	85 ± 2	[71]
F‐doped carbon	0.05 м H_2_SO_4_	– 0.3	N_2_、H_2_O	197.7 (µg mg^−1^_cat._ h^−1^)	54.8	[73]
h‐BNNs/CNTs	0.1 м Na_2_SO_4_	– 0.691	N_2_、H_2_O	36.5 (µg mg^−1^_cat._ h^−1^)	63.9	[74]
S_6.23_‐B_8.09_/CNFs	0.5 м K_2_SO_4_	– 0.7	N_2_、H_2_O	0.223 (µmol cm^−2^ h^−1^)	22.4	[75]

##### 2.2.2.2 Noble Metal Catalysts

In the research field of noble metal electrocatalysts for ammonia synthesis, researchers have constructed various high‐efficiency catalytic systems via different preparation methods and conducted in‐depth investigations into their performance and reaction mechanisms. McFarland and his colleagues successfully synthesized ruthenium (Ru) nanoparticles supported on carbon fiber paper using an oleate‐mediated method, and systematically studied their electrocatalytic performance for ammonia synthesis [76]. Electrochemical test results demonstrated that the Ru nanoparticles exhibited excellent catalytic performance in 0.1 м KOH electrolyte. Specifically, ammonia (NH_3_) yield rates of approximately 5.5 mg h^−1^ m^−2^ at 20°C and 21.4 mg h^−1^ m^−2^ at 60°C were achieved at a redox potential (E) of −100 mV versus the reversible hydrogen electrode (RHE). In contrast, a maximum Faradaic efficiency (FE) of about 5.4% was attainable at 10 mV vs. RHE.

Density functional theory (DFT) calculations revealed that nitrogen molecules can be effectively adsorbed at the edge sites of Ru (001) nanoparticles with a hexagonal close‐packed (HCP) structure, which is one of the key reasons for their high catalytic activity. Recently, Wang et al. developed an ultrafast reduction method to prepare a gold (Au) microstructure with a flower—like structure (Au Flower) and applied it to the reaction of nitrogen reduction [77].

In a 0.1 м HCl electrolyte, when the applied potential was – 0.2 V (vs. standard hydrogen electrode), the Au microstructure exhibited excellent catalytic performance: the NH_3_ generation rate was as high as 25.57 µg mg^−1^_cat._ h^−1^, and the Faraday efficiency reached 6.05%. The study suggested the highly dendritic structure of the flower—like structure can provide a large number of exposed active sites, thus significantly improving the catalytic reaction kinetics. Shi et al. prepared a series of Au—supported TiO_2_ catalysts and tested their N_2_ electro‐reduction in a 0.1 м HCl aqueous solution. Using tannic acid (TA) and sodium borohydride (NaBH_4_) as reducing agents and photoreduction method, respectively, the particle size of Au was successfully adjusted from 0.5 nm to 0.4 nm and 37 nm. The TA—reduced Au/TiO_2_ catalyst showed very high activity and selectivity for N_2_ electro—reduction, and the maximum ammonia generation rate reached 21.4 µg mg^−1^_cat._ h^−1^ (corresponding to 3.5 × 10^−10^ mol s^−1^ cm^−2^) with a Faraday efficiency of 8.11% at – 0.2 V vs RHE [79].

**FIGURE 5:**
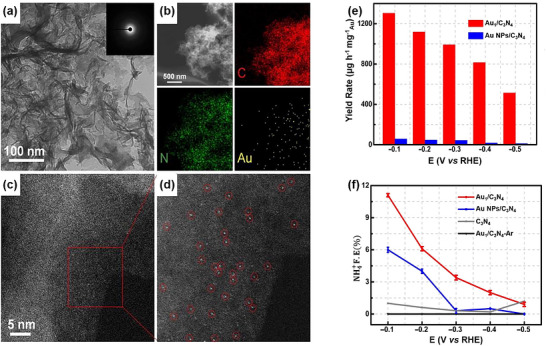
TEM observation of Au_1_/C_3_N_4_. (a) TEM images of Au_1_/C_3_N_4_ and corresponding SEAD pattern (inset). (b) Examination of the corresponding EDX mapping reveals the homogeneous distribution of Au, C and N on the nanosheets. (c), (d) Magnified HAADF‐STEM images of Au_1_/C_3_N_4_ directly show the atomic dispersion of Au atoms. (e) NH+ 4 yield rates normalized by Au mass at different potentials. (f) NH+ 4 formation Faradaic efficiencies for Au_1_/C_3_N_4_, Au NPs/C_3_N_4_, pure C_3_N_4_ and Au_1_/C_3_N_4_ with Ar feed instead of N_2_ at different potentials. Reproduced with permission [78]. Copyright 2026, Elsevier.

Wang et al. prepared atom—dispersed Au atoms on carbon nitride (Au_1_/C_3_N_4_) by reducing HAuCl_4_ with H_2_ at 40°C. Morphological testing shows that Au_1_/C_3_N_4_ retains the independent nanosheet structure of C_3_N_4_, and Au atoms are uniform throughout the entire nanosheets (Figure 5a–d). Compared with the Au nanoparticles supported on C_3_N_4_ (Au NPs/C_3_N_4_, Au particle size ≤ 8 nm), all Au atoms in Au_1_/C_3_N_4_ serve as exposed active sites, realizing 100% atomic utilization efficiency, while Au nanoparticles in Au NPs/C_3_N_4_ only have surface atoms available as active sites with low atomic utilization. Moreover, the Gibbs free energy of the rate‐determining step for Au_1_/C_3_N_4_ is merely 1.33 eV, much lower than the 2.01 eV of Au NPs/C_3_N_4_, making it easier to activate key intermediates. Thus, the maximum ammonia generation rate was 1305 µg mg^−1 Au^ h^−1^ in a N_2_ ‐ saturated 5 mм H_2_SO_4_ aqueous solution, and the Faraday efficiency of 2.8 × 10^−11^ mol s^−1^ cm^−2^ and 11.1% were achieved at – 0.1 V vs. RHE (Figure 5e,f) [78]. The utilization of low‐cost metals or metal‐free materials for the construction of catalytic systems, as well as the modification of the electronic structure of catalysts, may serve as an effective approach to minimize expenses, inhibit hydrogen evolution reaction (HER), and enhance nitrogen reduction reaction activity and selectivity in aqueous environments. These improvements provide significant insights and novel pathways for the industrialization of electrocatalytic ammonia production.

### 2.3 Other Catalytic Methods

Photocatalytic ammonia synthesis uses solar energy as a driving force, which is an abundant and cost‐free resource in most regions of the Earth, offering economic advantages for driving ammonia synthesis under mild conditions compared to other technologies [80]. In the photocatalytic ammonia synthesis process: first, photons serve as the driving force to excite electrons within the catalyst. Second, upon absorption of light energy, electrons are excited to the conduction band, leaving behind holes in the valence band. And then the dissociation of photogenerated charge carriers: some of the electrons and holes recombine, while the remaining electrons and holes migrate to the surface of the catalyst. Finally, these electrons and holes drive the redox reactions necessary for the process.

**FIGURE 6:**
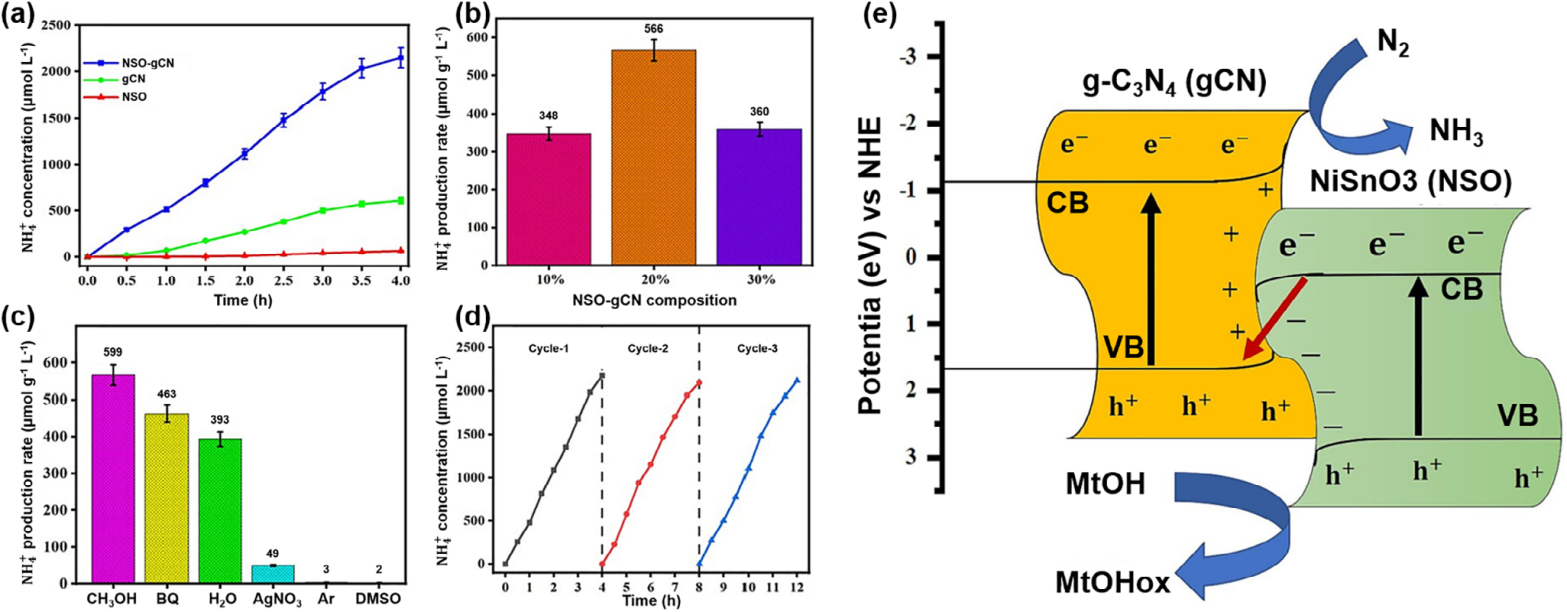
(a) Variation in concentration of photocatalytically generated NH+ 4 with time. (b) production rate of NSO‐gCN with different mass fractions of NSO. (c) production rate in the presence of scavengers (i.e. CH_3_OH, BQ, AgNO_3_), and without scavenger (H_2_O alone), DMSO and Ar. (d) recyclability test. (e) An S‐scheme electron transfer mechanism for the photocatalytic reaction over NSO‐gCN. Reproduced with permission [88]. Copyright 2026, Elsevier.

In 1977, Schrauzer and Guth pioneered the photoreduction of N_2_ using TiO_2_ photocatalyst. Since then, various semiconductors, including g‐C_3_N_4_, BiOBr, and metal oxides, have been utilized in research on photocatalytic ammonia synthesis [81, 82, 83, 84, 85, 86, 87]. However, the development of photocatalytic systems remains challenging due to several limitations, such as the restricted utilization of the visible light spectrum, rapid recombination of photogenerated electrons and holes, sluggish reaction kinetics, and high costs associated with many photocatalysts. Therefore, Extensive research has been conducted on the rational design and manufacture of photocatalysts with high cost‐effectiveness and structural stability. For example, the heterojunction formed by low‐cost nickel‐based perovskite (NiSnO_3_, NSO) and g‐C_3_N_4_ (gCN) nanosheets has recently demonstrated exceptional performance for photocatalytic ammonia synthesis under aqueous conditions [88]. After 5 h of reaction, an NH_3_ production rate of 566 µmol g^−1^ h^−1^ was achieved at room temperature (Figure 6a–d). The heterojunction structure of NSO‐gCN induces band bending at the interface between positively charged gCN and negatively charged NSO, forming an internal electric field directed from gCN to NSO (Figure 6e). Upon visible‐light irradiation, electrons are excited from the valence band (VB) to the conduction band (CB) of both NSO and gCN. Due to the influence of the internal electric field, electrons in the CB of NSO recombine with holes in the VB of gCN, leaving abundant electrons in the CB of gCN and holes in the VB of NSO, thus enhancing the photocatalytic activity for ammonia synthesis. Iron, characterized by its excellent stability, low cost, and favorable catalytic properties, has been extensively employed in thermal catalytic ammonia synthesis. However, iron‐based materials face limitations such as high electron‐hole recombination rates, low charge mobility, short hole diffusion lengths, and conduction band positions unfavorable for photocatalytic reduction reactions. One effective strategy to address these challenges involves doping iron into other semiconductors. Liu et al. doped Fe into BiOBr catalysts, altering its band structure to extend visible light absorption edges to 600 nm [89]. Additionally, Fe atoms connected to oxygen vacancies captured photogenerated electrons from nearby atoms, forming electron‐rich Fe(II) species that transferred surplus electrons to the anti‐bonding orbitals of N_2_. This strategy achieved a nitrogen fixation rate of 382.68 µmol g^−1^ h^−1^ at 25°C, with a surface‐area‐normalized ammonia generation rate of 5.74 µmol m^−2^ h^−1^. Plasma is regarded as the fourth state of matter, consisting of a conducting gas that is wholly or partially ionized. The generation process is as follows: gas is ionized by an external force, resulting in equal quantities of electrons and positively charged ions, along with the potential formation of free radicals. The electrons, particles, and free radicals will participate in a recombination reaction, ultimately resulting in the formation of atoms and molecules, rendering the system electrically neutral. Currently, numerous low‐temperature plasma catalytic techniques for ammonia synthesis exist, including dielectric barrier discharge (DBD), microwave discharge (MW), gliding arc (GA) discharge, glow discharge (GD), and radio frequency discharge (RF) [90, 91, 92]. Despite significant enhancements in ammonia synthesis yield via low‐temperature plasma under standard temperature and pressure, the yield and energy efficiency of this approach remain inferior to those of the Haber‐Bosch process. To address these issues, researchers have examined the plasma source, catalyst type, process parameters, and reaction mechanism to enhance the ammonia conversion rate.

The initial catalysts employed for ammonia synthesis under plasma conditions were iron and molybdenum wires. Tanaka et al. employed iron and molybdenum wires as catalysts to manufacture ammonia under circumstances of 650 Pa, utilizing radio frequency and microwave discharge [93]. The reaction temperature was 350°C, with radio frequency and microwave discharge frequencies of 13.56 MHz and 2540 MHz, respectively. The power was set at 180 W, and the continuous discharge duration lasted 2 h, resulting in NH_3_ concentration distributions of 1.1 mmol g^−1^ and 1.5 mmol g^−1^. Since the late 1990s, the synthesis of ammonia by atmospheric pressure packed dielectric barrier discharge (DBD) plasma catalysis has progressively emerged as a novel research avenue. Peng et al. synthesized a catalyst with Ru as the active component, with Cs, K, and Ba serving as promoters, utilizing the impregnation process. Experiments on ammonia synthesis were performed using DBD discharge at ambient pressure and low temperature, achieving a maximum energy efficiency of 2.41 g‐NH_3_ kWh ^−1^ [94]. Kim et al. employed Ru Mg/Al_2_O_3_ as a catalyst, integrating it with pulsed plasma to attain the energy efficiency of 35.7 g‐NH_3_ kWh^−1^ using the DBD method to date [95]. Wang et al. performed the reaction in a custom coaxial dielectric barrier discharge (DBD) plasma reactor with transition metals as catalysts (M/Al_2_O_3_, M = Fe, Ni, Cu) at around 35°C and atmospheric pressure [96]. In plasma catalysis, metal and weak acid sites create intermediates on the surface to facilitate NH_3_ production, while the synergistic interaction between transition metals and Al_2_O_3_ enhances the uniformity of plasma discharge capabilities. The NH_3_ synthesis rate of Ni/Al_2_O_3_ in the experiment attained a peak of 471 µmol g^−1^ h^−1^. Plasma can generate micro discharges or ephemeral substances within pores, or extend the longevity of a material in pores, hence facilitating plasma‐catalyzed ammonia production [97, 98]. Shah et al. produced ammonia using a 5A zeolite molecular sieve (Na*_x_* Ca*_y_* [(AlO_2_)_12_(SiO_2_)_12_]•*x*H_2_O)through dielectric barrier discharge plasma at atmospheric pressure [99]. This molecular sieve possesses an LTA structure, exceptional chemical stability, thermal stability, and uniform pores, hence mitigating potential mass transfer constraints in experiments. The experimental findings indicate that the zeolite molecular sieve enhances the micro discharge process, alters the voltage and current characteristics of the reactor, and induces substantial modifications in the collected external voltage charge waveform, consequently augmenting catalytic efficacy. The disruption of ambient plasma on the electrical characteristics of the zeolite surface may improve reaction states, facilitating nitrogen dissociation and ammonia production. With an equimolar nitrogen to hydrogen ratio, the energy yield in the presence of molecular sieves can attain 15.5 g‐NH_3_ kWh^−1^, which is a minimum of 50 times greater than that without molecular sieves.

Nitrogenases in nature catalyze ammonia synthesis under mild conditions, offering insights for the development of efficient and sustainable nitrogen fixation catalytic systems [100]. The iron‐containing protein in nitrogenase provides electrons to the MoFe protein by hydrolyzing adenosine triphosphate (ATP) into adenosine diphosphate (ADP) to facilitate the reduction of N_2_ [101]. Motivated by this, certain research initiatives have focused on creating catalysts with architectures akin to nitrogenase, with the objective of mimicking or replicating the functionality of nitrogenase. Brown et al. produced a molybdenum‐iron based nitrogenase protein from nitrogen‐fixing bacteria and successfully developed a biohybrid photocatalyst system by integrating it with highly photosensitive cadmium sulfide nanorods. Under environmental conditions, light‐excited CdS nanocrystals supply electrons to MoFe‐based proteins, facilitating the reduction of N_2_ to NH_3_. Under 405 nm light irradiation, the NH_3_ production rate of this system is 315 ± 55 nmol mg^−1^ min^−1^, with a turnover rate of 75 min^−1^, representing roughly 63% of the biological nitrogen fixation rate [102]. Kanatzidis et al. developed a method utilizing “Mo_2_Fe_6_S_8_‐SNH_3_S_6_” sulfur gel to substitute MoFe‐based proteins, wherein SNH_3_S_6_ sulfur gel serves as a light‐harvesting component, and Mo_2_Fe_6_S_8_ offers an active site for nitrogen reduction. Throughout the 72‐h testing duration, Mo_2_Fe_6_S_8_‐SNH_3_S_6_ sulfur gel exhibited remarkable photocatalytic stability [103]. Liu and colleagues subsequently developed a bionic sulfur gel system including [Mo_2_Fe_6_S_8_(SPh)_3_] and [Fe_4_S_4_] clusters [104]. Other inert ions, such as Sn^4+^ and Sb^3+^, can also impart photocatalytic activity for nitrogen reduction reaction to sulfur gel after substituting [Fe_4_S_4_] clusters. Ultimately, molybdenum‐free sulfur gel including solely Fe_4_S_4_ clusters can also facilitate the N_2_ fixation reaction, exhibiting even greater efficiency than the system incorporating Mo_2_Fe_6_S_8_(SPh)_3_. This signifies that whereas Mo_2_Fe_6_S_8_(SPh)_3_ is capable of fixing N_2_, Mo itself does not participate in this process. It is converted to NH_3_ in aqueous solution utilizing protons and sacrificial electrons. Nonetheless, the efficacy of these catalysts requires additional enhancement.

## 3 Ammonia Decomposition

### 3.1 Thermal Catalytic Ammonia Decomposition

#### 3.1.1 Synthesis Mechanism

The decomposition of ammonia represents a pivotal utilization pathway, primarily for the production of high‐purity hydrogen. Like ammonia synthesis, ammonia decomposition's reaction mechanism has been studied over an extended period of time. The following is an expression for the general chemical (Equation (2)):

(2) $$2NH_{3}\;⇌\;N_{2\;}+3H_{2},\;\Delta_{f}H_{0}=94.2\;\mathrm{kJ}\;\mathrm{mo}l^{-1}$$

The Ammonia decomposition reaction (ADR) is clearly an endothermic process with a volume increase, raising the temperature and lowering the pressure can thermodynamically favor the reaction. Lucentini's research indicating that the ammonia conversion rate surpasses 99% when ammonia breakdown is carried out at temperatures between 400°C and 900°C and reaction conditions of 1 bar [105].

Under these reaction conditions, Temkin et al. proposed that the pathway for ammonia decomposition is the inverse of that for ammonia synthesis. Initially, gaseous ammonia adsorbs onto the catalyst surface, followed by further dehydrogenation processes. Nitrogen and hydrogen atoms desorb and combine to generate N_2_ and H_2_. The precise response process is delineated as follows:

(3) $$N{H_{3}}_{\left(g\right)}+s\;⇌\;N{H_{3}}_{\left(\alpha \right)}$$

(4) $$N{H_{3}}_{\left(\alpha \right)}+s\;⇌\;N{H_{2}}_{\left(\alpha \right)}+H_{\left(\alpha \right)}$$

(5) $$N{H_{2}}_{\left(\alpha \right)}+s\;⇌\;NH_{\left(\alpha \right)}+H_{\left(\alpha \right)}$$

(6) $$NH_{\left(\alpha \right)}+s\;⇌\;N_{\left(\alpha \right)}+H_{\left(\alpha \right)}$$

(7) $$2N_{\left(\alpha \right)}\;⇌\;{N_{2}}_{\left(g\right)}+2s$$

(8) $$2H_{(\alpha )\;}⇌\;{H_{2}}_{\left(g\right)}+2s$$

In this context, the notation denotes species adsorbed on the catalyst surface, while “s” signifies unoccupied spaces on the catalyst surface. Investigations have been undertaken regarding the rate‐limiting step in the ammonia decomposition reaction. Love et al. conducted the first experimental study on the kinetics of ammonia decomposition, revealing that for a Fe‐K_2_O‐Al_2_O_3_ catalyst, the decomposition rate displayed a linear positive correlation with temperature (347°C–430°C) and was directly proportional to the partial pressure of hydrogen while being inversely proportional to the partial pressure of ammonia [106]. The nitrogen desorption process (Equation (7)) was identified as the rate‐limiting step. Conversely, for the two additional catalysts (Fe/Al_2_O_3_ and Fe catalyst), the reaction rate exhibited a positive correlation solely with temperature, while the rate‐limiting step was identified as the cleavage of the N‐H bond (Equations (5) and (6)). Logan and Kemball investigated the reaction rates of various catalysts and their dependence on ammonia and hydrogen pressures, concluding that the desorption of N_2_ from the catalyst surface was the rate‐limiting step for the examined catalysts (Ni, Co, Rh, Pt, Ru, Re, Fe, VN, and W) [107]. Subsequently, Takezawa and Toyoshima conducted an investigation utilizing a Fe catalyst enhanced with Al_2_O_3_, K_2_O, and SiO_2_, discovering that the ammonia decomposition rate grew with increasing reaction temperature, and the rate‐limiting step shifted from nitrogen desorption to the dehydrogenation of NH_2_ on the catalyst surface (Equation (5)) [108]. Generally, while the specific rate‐determining steps in the ammonia decomposition process depend on the catalyst composition and reaction conditions, the cleavage of the N‐H bond on the catalyst surface or the desorption of N_2_ is more likely to be the rate‐limiting step.

Presently, there are two application situations for ammonia decomposition. The dominant method is the generation of high‐purity hydrogen, which requires conducting the reaction under the mildest conditions feasible to guarantee cost efficiency. Another developing use is to the partial breakdown of ammonia (30 ‐ 50%) under elevated pressure. The products are designed for utilization in ammonia‐hydrogen engines, fuel cells, and similar applications. Notwithstanding thermodynamic limitations, employing high‐pressure hydrogen can circumvent the supplementary expenses linked to additional compression procedures.

Nowadays, ruthenium (Ru) catalysts are acknowledged as the most effective catalytic systems for low‐temperature NH_3_ breakdown and have been extensively studied among the ADR catalysts. This discussion will examine the effects of supports, promoters, and the size and morphology of ruthenium particles on the efficacy of ruthenium catalysts, and introduce other metals such as Ni and Co [109, 110, 111].

#### 3.1.2 Catalysts

##### 3.1.2.1 Noble Metal Catalysts

**FIGURE 7:**
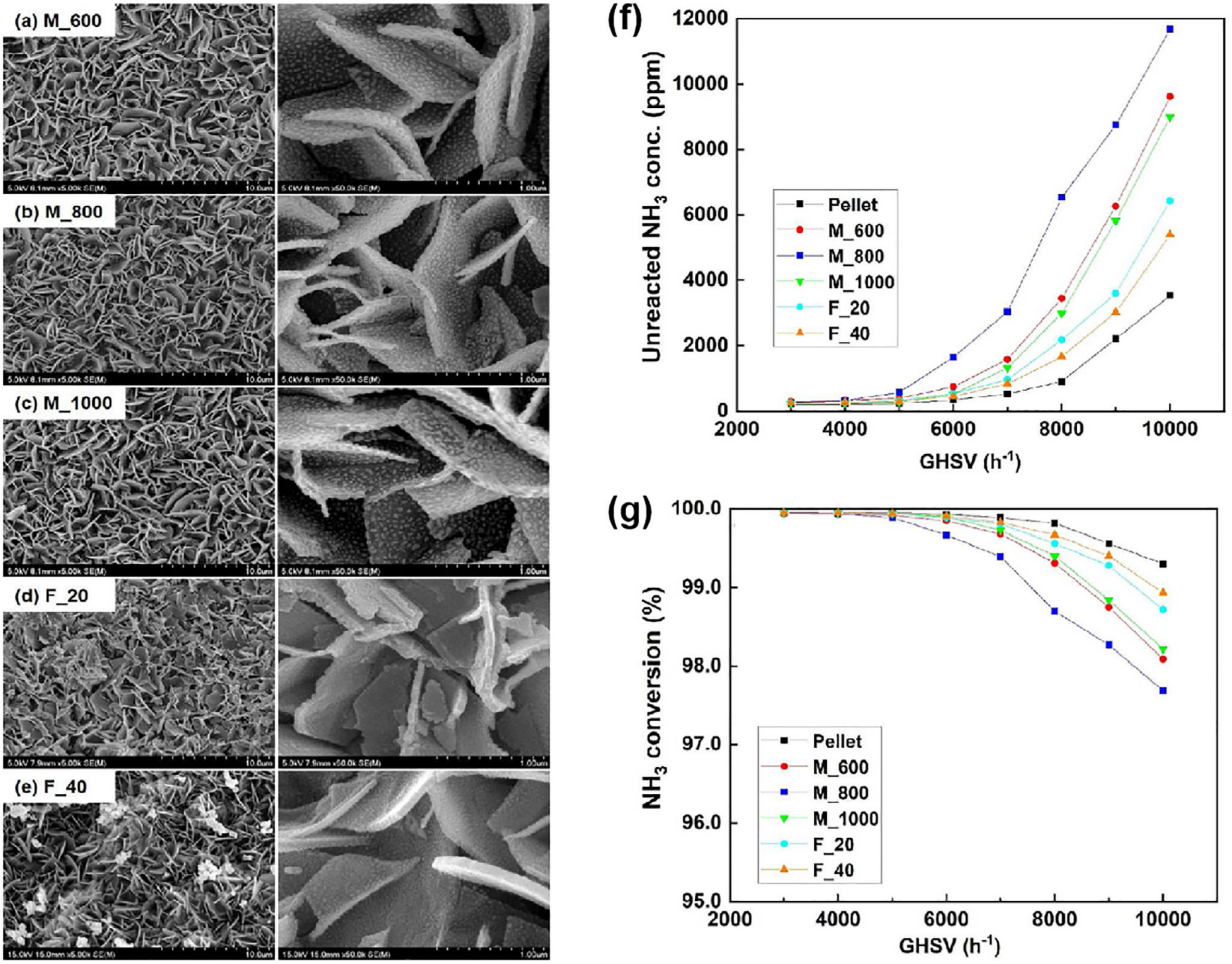
SEM images of Ru/Mg‐Al oxide catalyst layer coated on the FeCralloy metal substrate (a) M_600, (b) M_800, (c) M_1000, (d) F_20 and (e) F_40. Comparison of (f) unreacted NH_3_ concentration and (g) NH_3_ conversion of metal structured catalysts for different GHSVs in NH_3_ decomposition reaction (reaction condition: *T* = 700°C, GHSV = 3000—10 000 h^−1^). Reproduced with permission [116]. Copyright 2026, Elsevier.

Yin et al. investigated the catalytic activity of Ru‐loaded oxide/carbon supports in the study of ruthenium‐based catalysts and identified the following activity sequence: carbon nanotubes > MgO > TiO_2_> Al_2_O_3_> ZrO_2_> activated carbon [112]. The higher effectiveness of carbon nanotubes as a support is ascribed to the improved dispersion of Ru and their high electronic conductivity, which promote the desorption of N_2_ from the catalyst. Li et al. investigated various carbon materials as supports for Ru and determined that the catalytic activity of the Ru catalysts ranked as follows: graphitic carbon > carbon nanotubes > carbon black > mesoporous carbon CMK‐3 > activated carbon [113]. Their proposal indicates that an increased level of graphitization in carbon supports enhances electron transport, resulting in elevated turnover frequencies (TOF) for the associated catalysts. Duan et al. performed a study on ruthenium catalysts supported by carbon nanofibers (CNF) and carbon nanotubes (CNT). It was noted that for Ru crystals of identical dimensions, the ammonia decomposition conversion rate was significantly higher when Ru was supported on CNF rather than on CNT [114]. Additionally, Marco et al. employed CNF as a substrate for Ru and discovered that nitrogen doping of the substrate enhanced catalytic activity relative to undoped substrates [115]. In summary, both the content and form of the support significantly affect the activity of ruthenium‐based catalysts. Ruthenium‐based catalysts supported by nitrogen‐doped carbon nanotubes and carbon nanofibers demonstrate exceptional catalytic activity. Recently, Jung et al. examined Ru metal‐structured catalysts using various support architectures [116]. The team utilized FeCr alloys as supports, comprising monolithic support catalysts (e.g., M_600, M_1000) and foam support catalysts (e.g., F_20, F_40). The surfaces of these supports were coated with a high‐surface‐area MgAlO_x_ oxide layer obtained from the pyrolysis of layered double hydroxides (LDH). Subsequently, Ru nanoparticles were deposited onto the oxide layer surface using a precipitation method. This method improved the distribution of Ru nanoparticles, augmented the density of active sites, and decreased the Ru loading to one‐tenth of that in traditional pellet catalysts (Figure 7a–e). Figure 7f,g shows that monolithic catalysts demonstrated exceptional performance at low temperatures (≤650°C) and low gas hourly space velocities (GHSV = 3000 h^−1^), attaining ammonia decomposition efficiencies beyond 95%. Conversely, foam catalysts exhibited enhanced efficacy at elevated temperatures (>650°C) and high GHSV (>6000 h^−1^). Furthermore, the foam catalyst demonstrated steady long‐term operational efficacy in a hydrogen generation system, sustaining an ammonia conversion rate surpassing 99.0% over 100 h of operation. This indicates favorable opportunities for the development of compact and efficient ammonia decomposition reactors. In terms of promoters, wang et al. [117]. carried out activity tests on catalysts modified with different metal nitrates, revealing an activity sequence of K‐Ru > Na‐Ru > Li‐Ru > Ce‐Ru > Ba‐Ru > La‐Ru > Ca‐Ru > Ru, which indicates that increased electronegativity of the promoter correlates with a decreased NH_3_ conversion rate. Li et al. [118]. reported a relative activity sequence of K > Na > Ca > Cl > SO_4_ > PO_4_ > Li. Furthermore, Pyrz et al. [119]. synthesized catalysts employing various combinations of promoters. They formulated Ru/Al_2_O_3_ catalysts enhanced with Ba and K or Cs and K. At 350°C, the ammonia decomposition rate of the Ba and K co‐promoted Ru/Al_2_O_3_ catalyst was inferior to that of the K‐promoted Ru/Al_2_O_3_ catalyst. Dimensions and morphology of ruthenium (Ru) particles are essential determinants affecting catalytic efficacy. Researchers argue that the elevated activity of Ru (ruthenium) particles predominantly arises from the B5 sites on their surfaces. The B5 sites consist of single‐atom steps on the Ru (0001) crystal plane, characterized by three Ru atoms organized on the bottom layer and two Ru atoms accurately positioned on the adjacent higher layer. The minimal Ru particle size required to host B5 sites is hypothesized to be approximately 2 nm, but the size range exhibiting the greatest density of B5 sites is between 3 and 5 nm. Comprehensive research has demonstrated that the existence of B5 sites correlates not only with particle size but also with particle morphology [120]. Studies indicate that the optimal turnover frequency (TOF) values for hemispherical Ru particle catalysts are observed within the size range of 1.8–3 nm. Conversely, flat and elongated Ru particle samples demonstrate optimal activity at sizes of roughly 7 nm.

##### 3.1.2.2 Non‐Noble Metal Catalysts

**FIGURE 8:**
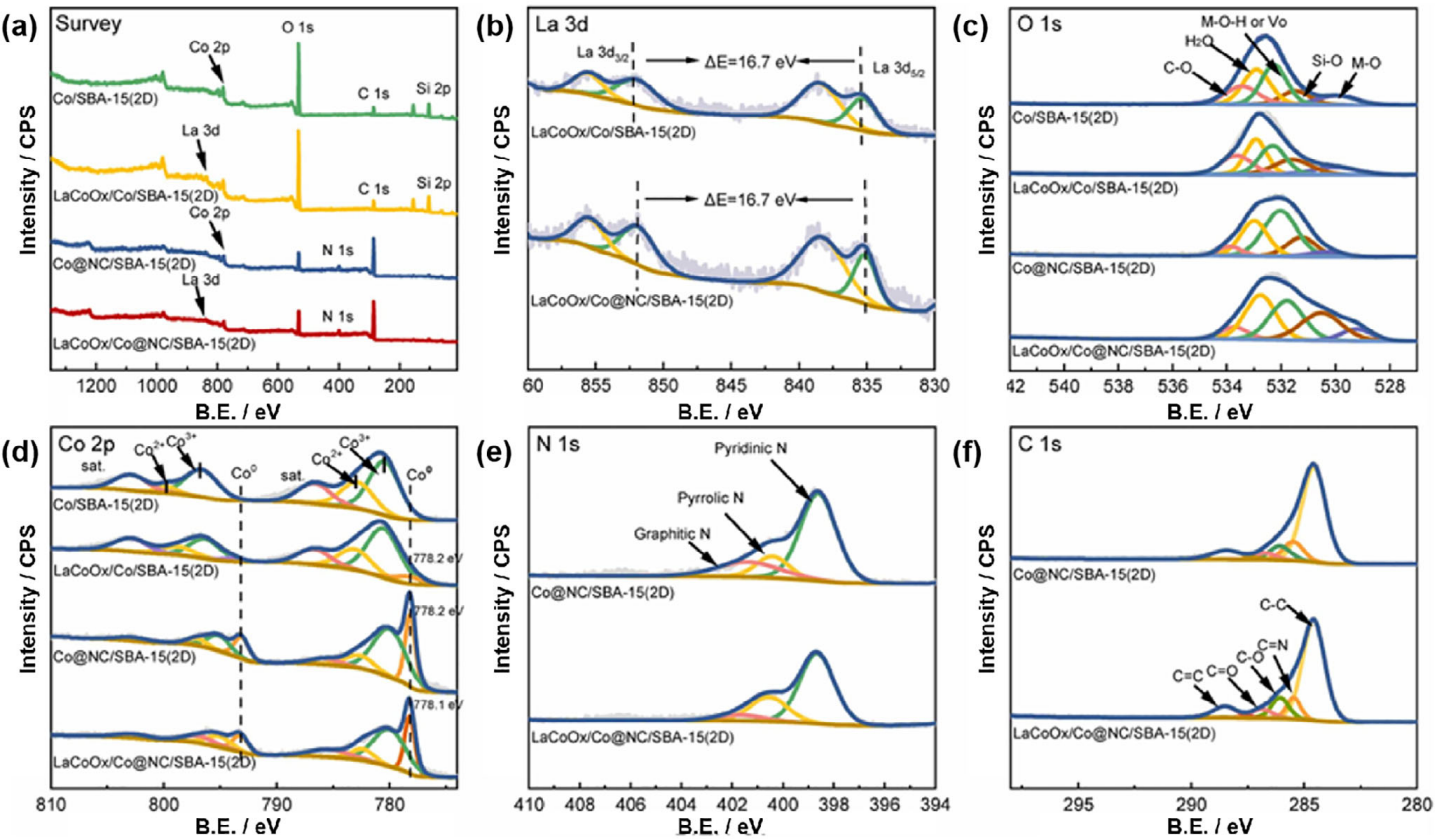
(a) XPS survey spectra of LaCoO_x_/Co@NC/SBA‐15(2D), Co@NC/SBA‐15(2D), LaCoO_x_/Co/SBA‐15(2D), and Co/SBA‐15(2D) catalysts. High‐resolution XPS spectra of LaCoO_x_/Co@NC/SBA‐15(2D), Co@NC/SBA‐15(2D), LaCoO_x_/Co/SBA‐15(2D), and Co/SBA‐15(2D) for (b)La, (c) O, (d) Co, (e) N and (f) C. Reproduced with permission [122]. Copyright 2026, Elsevier.

Ru‐based catalysts are challenging to use in industrial production due to their elevated cost, prompting the investigation of alternative materials (such as Co, Ni, etc.) to substitute Ru‐based catalysts, with notable advancements occurring in this domain in recent years [121, 122, 123, 124, 125, 126]. For instance, Han et al. synthesized a dual‐spatially confined catalyst, LaCoO_x_/Co@NC/SBA‐15(2D), within the Co‐based catalyst framework using in situ restricted pyrolysis of nano‐La‐doped ZIF‐67 within the mesopores of SBA‐15(2D) [122]. This catalyst combines LaCoO_x_‐modified Co nanoparticles within the NC matrix and two‐dimensional mesoporous silica. Among these, The La 3*d* spectra confirm the formation of amorphous LaCoO_x_ species, while the O 1*s* spectra reveal abundant surface oxygen vacancies and defect sites. The Co 2p spectra indicate the coexistence of Co^0^, Co^2+^, and Co^3+^, and the introduction of LaCoO_x_ modulates the electronic structure of Co, promoting electron transfer to Co^0^ and thereby enhancing the capability for N‐H bond activation. Meanwhile, the N 1*s* and C 1*s* spectra verify the successful doping of nitrogen into the carbon matrix, forming various bonding configurations such as pyridinic N, pyrrolic N, and graphitic N. These not only stabilize the Co nanoparticles and suppress their aggregation but also optimize charge transport through strong interactions. (Figure 8a–f). This catalyst attains complete ammonia breakdown at 550°C and a gas hourly space velocity (GHSV) of 30 000 g^−1^_cat._ h^−1^, achieving a hydrogen production rate of 446 mmol g^−1^ Co min^−1^ over 200 h at 600°C and a GHSV of 60 000 g^−1^_cat._ h^−1^, surpassing the performance of most documented Co‐based catalysts. Lanthanide compounds (e.g., La_2_O_3_, LaCoO_x_) utilized as supports can augment basicity, increase oxygen vacancy concentration, and enhance electron density at active sites, so markedly enhancing the efficacy of catalytic systems. This has attracted significant attention from numerous experts [127, 128, 129]. For instance, Yin et al. similarly investigated the function of lanthanide compounds in nickel‐based systems, synthesized a series of Ni nanoparticle catalysts (Ni_6_Al_2_LaO_x_) tightly anchored onto an Al_2_O_3_‐La_2_O_3_ composite support, using layered double hydroxides (LDHs) as precursors. The Ni_6_Al_2_LaO_x_ catalyst attained a hydrogen generation rate of 30.47 mmol g^−1^_cat._ min^−1^ at 600°C with a weight hourly space velocity (WHSV) of 30 000 mL g^−1^_cat._ h^−1^. Furthermore, following a 40‐h stability assessment characterized by cyclic temperature fluctuations (500°C → 550°C → 500°C), the catalyst demonstrated a deactivation rate of < 1%, signifying considerable promise for industrial utilization [130].

In general, the hydrogen‐rich gas generated from NH_3_ decomposition contains residual ammonia and a certain percentage of nitrogen, necessitating purification prior to utilization. Widely employed industrial purification techniques encompass physical methods such as pressure swing adsorption (PSA), membrane separation, and cryogenic separation, with chemical methods including CO preferential oxidation (COPROX), CO selective methanation, and metal hydride separation. In the ammonia breakdown process for hydrogen synthesis, the resultant hydrogen‐rich gas is devoid of CO_x_, allowing for separation using physical means. When handling substantial quantities, techniques such as PSA and cryogenic separation may be utilized. Conversely, membrane separation can enhance large‐scale separation technologies like as PSA by mitigating challenges related to extensive footprint and intricate maintenance, so rendering it appropriate for processing smaller batches [131, 132].

### 3.2 Photocatalytic Ammonia Decomposition

#### 3.2.1 Synthesis Mechanism

Photocatalytic ammonia decomposition has emerged as a promising alternative route for hydrogen production from ammonia. Since the pioneering report by Fujishima and Honda on photoelectrochemical water splitting in 1972, photocatalysis has been extensively applied to a variety of chemical reactions [133].  Inspired by these developments, numerous studies have been devoted to driving ammonia decomposition under light irradiation [134, 135, 136, 137].

Photocatalytic ammonia decomposition for hydrogen production proceeds as follows: When the incident photon energy exceeds the bandgap of the photocatalyst, electron‐hole pairs are photogenerated. Under such conditions, photogenerated holes (h^+^) in the valence band (VB) can oxidize ammonia molecules to nitrogen (N_2_) and hydrogen ions (H^+^), while photogenerated electrons (e^−^) in the conduction band (CB) subsequently reduce H+ to molecular hydrogen (H_2_) (Equations (9)–(11)).

(9) $$\mathrm{phtotcatalyst}\;\to \;h^{+}+e^{-}$$

(10) $$2NH_{3}+6h^{+}\;\to \;N_{2}+6H^{+}$$

(11) $$6H^{+}+e^{-}\;\to \;3H_{2}$$

#### 3.2.2 Catalysts

##### 3.2.2.1 Noble Metal Catalysts

Li et al. constructed a Ru nanoparticle‐decorated GaN nanowire/Si (Ru NPs/GaN NWs/Si) photothermal‐coupled catalytic architecture in this study, which exhibits distinct structural advantages. The architecture features vertically aligned GaN nanowires with a length of ∼950 nm and an average diameter of ∼50 nm grown on a silicon wafer via molecular beam epitaxy, onto which Ru NPs with an average size of ∼19 nm were uniformly deposited. Such a well‐defined one‐dimensional nanostructure not only offers a high surface area and shortened charge diffusion pathway but also enables efficient utilization of the full solar spectrum through the pronounced photothermal effect of the Si substrate. XPS analysis reveals electron redistribution between Ru and GaN, and Bader charge calculations further confirm charge transfer from Ru to GaN, which contributes to the stabilization of active sites and enhanced interfacial electron transport. In terms of catalytic performance, this system achieves a hydrogen evolution rate of 3.98 mmol cm^−2^ h^−1^ under full‐spectrum illumination at 5 W cm^−2^, representing an approximately 1000‐fold enhancement compared to purely thermal catalysis at the same temperature. The apparent activation energy for ammonia decomposition decreases markedly from 1.08 eV to 0.22 eV under light irradiation, demonstrating the synergistic contribution of photogenerated charge carriers and the photothermal effect. Wavelength‐controlled experiments further verify that the reaction is efficiently driven by the combined effects of charge carriers generated from UV light and thermal energy contributed by visible/infrared light. Moreover, the catalyst maintains excellent stability over 400 h of continuous illumination, delivering a high turnover number (TON) of 3 400 750 mol H_2_ per mol Ru, which underscores its outstanding durability [138]. The work by Halas et al. has designed a Cu‐Ru antenna‐reactor (Cu‐Ru‐AR) structure, which enables efficient ammonia splitting under light illumination. The active sites in Cu‐Ru‐AR consist of Cu, which exhibits strong localized surface plasmon resonance (LSPR), and Ru, known for its excellent thermal catalytic activity. Upon illumination, hot carriers are efficiently transferred from Cu to Ru, thereby activating the ammonia molecules adsorbed on the Ru sites [139, 140].

##### 3.2.2.2 Non‐Noble Metal Catalysts

TiO_2_ is one of the most widely employed photocatalysts due to its competent performance in diverse applications. These encompass energy‐related reactions (e.g., water splitting, hydrogen production, CO_2_ reduction, nitrogen fixation), environmental remediation (e.g., nitrogen oxide reduction, pollutant degradation), and chemical synthesis (e.g., organic reactions, ammonia synthesis and decomposition) [141]. TiO_2_ suffers from a low light‐harvesting rate and rapid recombination of photogenerated charge carriers. However, it has been reported that the introduction of co‐catalysts or dopants can significantly enhance its photocatalytic performance by extending light absorption, optimizing the adsorption of reactants or products, and improving charge‐carrier separation efficiency [142, 143, 144].

For instance, Utsunomiya et al. compared the photocatalytic activity for ammonia decomposition under UV irradiation at room temperature using TiO_2_ loaded with various metals, the Ni/TiO_2_ photocatalyst demonstrated a peak H_2_ generation rate of 131.7 µmol g^−1^_cat._ [145]. Furthermore, TiO_2_ nanoparticles supported on light expanded clay aggregate (LECA) particles also demonstrate enhanced photocatalytic activity for ammonia decomposition. Under optimized conditions (calcination temperature: 550°C; pH: 11.0), this system achieved over 85% ammonia removal within 300 min [146]. In parallel, Obata et al. investigated a series of Pt‐loaded, metal‐doped TiO_2_ (Pt/M‐TiO_2_) photocatalysts for the decomposition of NH_3_ into H_2_ and N_2_ under UV irradiation at room temperature. Among them, the Fe‐doped catalyst (Pt/Fe‐TiO_2_) yielded the highest amount of H_2_. This superior performance is attributed to the substitution of Ti^4+^ sites by Fe^3+^ ions, which does not alter the fundamental TiO_2_ crystal structure but effectively shifts its absorption edge from the UV to the visible light region [147].

Similar to TiO_2_, ZnO is also regarded as one of the most widely used photocatalysts due to its high efficiency, low cost, and environmental sustainability [148, 149]. Reli et al. reported that ZnO prepared by thermal annealing of zinc acetate in air exhibited a high hydrogen yield, with activity even surpassing that of commercial TiO_2_ Evonik P25. This enhanced performance was attributed to the low concentration of oxygen vacancies in the synthesized ZnO, which effectively suppressed the recombination of photogenerated electron‐hole pairs [150]. In a related study, Mohammadi et al. constructed a heterostructure by immobilizing TiO_2_/ZnO on lightweight expanded clay aggregate (LECA) for ammonia removal from synthetic wastewater. The efficiency of ammonia decomposition was significantly influenced by the mobility and lifetime of charge carriers generated in the TiO_2_/ZnO composite [151]. As a result, an ammonia removal rate of 95.2% was achieved after 3 h of UV irradiation. Guo et al. synthesized a porous Ag‐loaded ZnO heterogeneous photocatalyst by a one‐pot method, and the interface contact and the electron transfer capacity between ZnO and other materials were proved to be enhanced [152]. The photocatalytic activity for decomposition of ammonia was significantly improved after Ag modify cation and retained high efficiency in the stability experiment; this can be attributed to inhibition of the recombination process of photogenerated electrons and holes by interconversion between Ag^+^ and Ag0 at the surface of ZnO. In addition, the effective separation of the photogenerated carriers can generate more active groups, which can promote the degradation of ammonia.

### 3.3 Other Catalytic Methods

In recent decades, research in this domain has mostly focused on developing ways for ammonia decomposition at lower temperatures, with the goal of reducing the process's operational costs and energy needs. Currently, recent ammonia decomposition technologies such as photocatalysis, and plasma catalysis show significant benefits, allowing reactions to take place at lower temperatures or using renewable energy sources. For instance, the catalytic activity of ammonia breakdown can be greatly increased by the electrocatalytic system. Lim et al. used a battery based on cesium dihydrogen phosphate (CDP), a proton conducting electrolyte that can produce hydrogen gas at a relatively fast rate when combined with ammonia's thermal breakdown. Plasma heating can additionally augment catalytic activity [153]. El‐Shafie et al. attained peak NH_3_ conversion rates of 85% and 84% utilizing Ru/Al_2_O_3_ and SiO_2_ catalysts, respectively [154]. Microwave‐assisted catalysis provides enhanced heating efficiency relative to conventional approaches. In the Co_2_Mo_3_N process at 400°C, the ammonia conversion rate surpassed 90%, with an activation energy of 31 kJ mol^−1^, and the energy efficiency was 90 times greater than that of traditional thermally driven processes [155].

## 4 Summary and Outlook

This work focuses on the ammonia‐hydrogen energy conversion and systematically reviews the technological status and core breakthroughs of ammonia synthesis and ammonia decomposition under mild conditions through thermal catalysis, electrocatalysis, photocatalysis and so on. At present, many advancements have been made in this field. However, there is still a long way to go from the laboratory to industrial application, where are still many significant and challenging drawbacks. First, both the reaction pathways of ammonia synthesis and ammonia decomposition involve multiple electron transfers and complex reaction intermediates. The adsorption and desorption behaviors on different metal surfaces are different, and the rate‐limiting steps remain unclear. Combining theoretical calculations including density functional theory (DFT) and molecular dynamics (MD) with in situ characterizations could reveal the complex mechanisms of ammonia synthesis and decomposition, thereby providing guidance for the rational design of catalysts. Second, noble metal catalysts exhibit superior performance compared to non‐noble metal catalysts in both ammonia decomposition and ammonia synthesis. Thus, designing low‐cost and efficient catalysts could promote the industrialization of this field. Meanwhile, the catalysts are often poisoned by specific pollutants, which would also affect the long‐term stability in actual processes. Based on the above considerations, high‐throughput screening of catalysts via machine learning (ML) combined with the adoption of non‐noble metals, improved atomic utilization efficiency of noble metals, and design of low‐cost composite supports with high specific surface area and strong metal‐support interaction to enhance the dispersion and structural anchoring of active sites, as well as the introduction of alkali/alkaline‐earth/transition metal composite promoters to realize multi‐effect synergistic regulation through the modulation of acid‐base sites and electronic configurations, collectively provides robust guidance for the development of advanced catalysts with a balanced integration of high activity, excellent stability and low cost under practical operating conditions. Finally, a complete ammonia‐hydrogen energy conversion technology encompasses upstream hydrogen production, green ammonia synthesis, liquid ammonia transportation and terminal applications. And each process includes basic units such as raw material acquisition, manufacturing, operation, and scrap recycling. Building a dynamic evaluation system including life cycle assessment (LCA) and techno‐economic analysis (TEA) could quantify the real environmental benefits of the technology.

## Conflicts of Interest

The authors declare no conflicts of interest.

## Data Availability

Data availability is not applicable to this article as no new data were created or analyzed in this study.
